# NLRP6 as a candidate segmental barrier to carcinogenesis in the human gut: a hypothesis

**DOI:** 10.3389/fimmu.2026.1823134

**Published:** 2026-08-05

**Authors:** Ginés Luengo-Gil, Ana Belén Arroyo, Ana María Hurtado-López, José García-Solano, Pablo Conesa-Zamora, Ana Tapia-Abellán

**Affiliations:** 1 Group of Molecular Pathology and Pharmacogenetics, Pathology Department, Instituto Murciano de Investigación Biosanitaria (IMIB)-Pascual Parrilla, Hospital General Universitario Santa Lucía, Cartagena, Spain; 2 Health Sciences Faculty, Universidad Católica de Murcia (UCAM), Murcia, Spain; 3 Group of Immunology, Department of Biochemistry and Molecular Biology (B) and Immunology, Campus de Ciencias de la Salud, Universidad de Murcia, Murcia, Spain

**Keywords:** colorectal cancer, NLRP6, inflammasome, human gut, mucosal immune surveillance, tumour microenvironment

## Abstract

Colorectal cancer (CRC) is a major global health burden, whereas small intestinal cancers are rare despite arising within the same gastrointestinal tract. This disparity suggests that segment-specific epithelial programmes may influence carcinogenesis. NLRP6 is an inflammasome-forming sensor with established roles in epithelial homeostasis, barrier maintenance, mucosal immune surveillance, and host–microbiota interactions. Public human tissue resources, an exploratory reanalysis of currently available single-cell data, and our local colonic cohort, provide hypothesis-generating observations compatible with segmental differences in NLRP6 expression, including very low bulk transcript abundance in human colonic samples. However, the currently available human evidence remains limited and heterogeneous, and does not yet support a simple binary model of presence in the small intestine and absence from the colon. Murine studies nevertheless support biological plausibility, as Nlrp6 deficiency exacerbates inflammation-driven colonic tumourigenesis and impairs epithelial repair. In addition, independent human studies indicate protein-level and clinicopathological relevance of NLRP6 in colonic disease, suggesting that its expression may vary according to segment, cell type, inflammatory context, and disease state. We therefore propose that NLRP6 may function as one candidate component of a segment-restricted epithelial defence and mucosal immune surveillance network in the human gut, potentially shaping CRC-relevant inflammatory and barrier conditions rather than acting as an established human tumour-suppressive mechanism. This model should now be tested through orthogonal human validation, including protein-based assays, spatial and single-cell analyses, and correlation with inflammatory status and clinical outcome, before any biomarker-guided intervention strategies are considered.

## Introduction

Colorectal cancer (CRC) is the third most commonly diagnosed malignancy worldwide, accounting for 10% of all cancer cases, with 1.9 million new cases in 2022, according to GLOBOCAN data (1). It is the second leading cause of cancer-related deaths globally, causing over 900, 000 deaths annually, despite effective screening. More than 5 million people live within five years of CRC diagnosis, indicating its significant global prevalence. Geographical disparities persist, with the highest incidence in high- and upper-middle-income countries, along with rising early onset disease. In contrast, small intestinal cancers are rare, representing less than 1% of all new cancer cases worldwide. GLOBOCAN estimates suggest 60, 000–70, 000 new cases globally in 2020, corresponding to an age-standardised incidence of 0.6 per 100, 000 person-years. Although less frequent than colorectal cancer, small intestine tumours significantly impact gastrointestinal cancer mortality; they account for 0.2–0.3% of cancer deaths, with 13, 000–15, 000 deaths yearly and age-adjusted mortality rates of 0.3–0.4 per 100, 000 in high-income settings (1). The five-year prevalence remains low compared to other digestive cancers but rises with increasing incidence, particularly among older adults and in high-income countries, where neuroendocrine histologies predominate. Despite arising within the same gastrointestinal continuum, colorectal and small intestinal cancers differ epidemiologically. While CRC poses a major global health burden, small intestinal malignancies remain rare. This contrast reflects the distinct biological, environmental, and diagnostic factors that shape the risk of cancer across the digestive tract. Several mechanisms can explain these differences. The small intestine has more fluid and alkaline content, faster transit, and lower exposure to dietary carcinogens (2), limiting mutagen contact. Its bacterial load is lower than that of the colon, with microbiota profiles generating fewer carcinogenic bile acids and genotoxins (3, 4). The small intestinal mucosa exhibits intense epithelial turnover and robust IgA-rich mucosal immunity (5, 6), which enhances transformation clearance. Regional differences in stem cell dynamics and Wnt signalling (7, 8), with distinct genetic and environmental risk profiles, create conditions that are less conducive to malignant transformation in the small intestine.

Epidemiological, microbiological, and mucosal immunology data show significant differences between the small intestine and colorectum; however, they do not fully explain the disparities in cancer incidence and mortality. Models often overlook segment-specific epithelial programmes (9), complicating observations such as reduced colorectal cancer risk with thiazolidinedione use. These gaps suggest undiscovered tract-segment-restricted mechanisms, providing a basis for testing an integrative hypothesis in future studies. Within this multifactorial context, epithelial innate immune pathways may represent underexplored determinants of regional intestinal cancer susceptibility. Among these, NLRP6 emerges as a candidate epithelial defence component rather than as a solitary explanation for regional differences in intestinal carcinogenesis.

NLRP6 is a cytosolic sensor of the NOD-like receptor family that has been widely implicated in intestinal homeostasis. Upon activation, NLRP6 oligomerises and recruits the adaptor protein ASC, which in turn engages caspase-1 to assemble an inflammasome complex. This complex mediates the processing and release of pro-inflammatory cytokines such as IL-1β and IL-18 and can induce inflammatory cell death through GSDMD cleavage and pyroptosis, thereby linking microbial and damage sensing to epithelial defence (5, 10). In the mouse gut, Nlrp6 has been described in intestinal epithelial and goblet-cell compartments and linked to IL-18 maturation, mucus secretion, epithelial renewal, and host–microbiota interactions (3, 4, 7, 11, 12). Recent mechanistic work in human and mouse intestinal epithelial cells further suggests that NLRP6 assembles an inflammasome in response to sterile or pathogen-induced endolysosomal damage, leading to canonical inflammasome activation (13). This study also suggests that, at least in this epithelial context, NLRP6 activation may rely more on endolysosomal damage sensing than on previously proposed direct activation by pathogen-associated ligands such as dsRNA or lipoteichoic acid (13). Taken together, these observations suggest that regional susceptibility to malignancy across the gastrointestinal tract may depend not only on differences in luminal exposure, microbiota composition, or stem-cell behaviour, but also on underexplored segment-specific epithelial defence programmes. Among several candidate epithelial defence pathways, NLRP6 is of particular interest because it may integrate barrier maintenance, inflammasome signalling, epithelial renewal, and responses to microbial or sterile injury. Although much of the mechanistic literature derives from murine systems, emerging human data indicate a more restricted and potentially segmental expression pattern, raising the possibility that NLRP6 biology may contribute differently across intestinal regions. In this framework, NLRP6-mediated epithelial defence can be interpreted not only as a barrier-preserving programme, but also as a component of mucosal immune surveillance that may influence tumour initiation by shaping IL-18-dependent signalling, inflammatory tone, microbiota–host interactions, and the local tumour-permissive or tumour-restraining microenvironment.

## Hypothesis

Based on converging but still incomplete lines of evidence, we propose that NLRP6 may represent a candidate component of a segment-specific epithelial defence programme in the human gut, potentially exerting a stronger protective role in the small intestine than in the colorectum and thereby contributing to regional differences in susceptibility to intestinal carcinogenesis.

### Human observations compatible with a hypothesis of segmental NLRP6 enrichment

**Figure 1:**
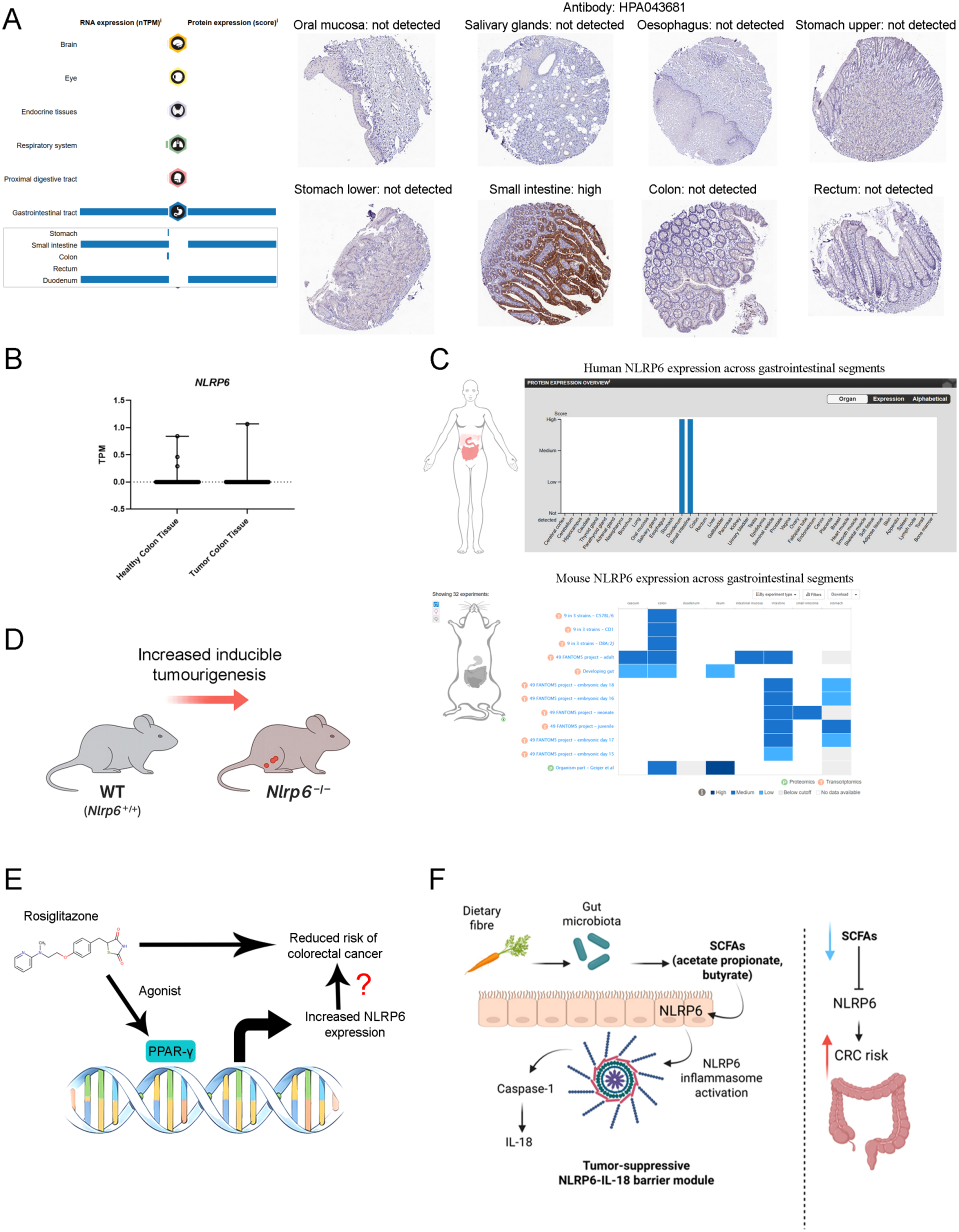
Observations and hypotheses supporting a candidate segment-restricted NLRP6 model in the gut. **(A)** Illustrative human NLRP6 RNA/protein overview and representative immunohistochemistry adapted from The Human Protein Atlas (https://www.proteinatlas.org/ENSG00000174885-NLRP6/tissue), antibody HPA043681 (SigmaAldrich; accessed December 12, 2025), shown as a public tissue-expression resource supporting the hypothesis, but requiring orthogonal validation for definitive protein-level interpretation. **(B)** NLRP6 mRNA expression (TPM) in our human colonic cohort, comparing healthy colon with tumour tissue, showing very low bulk transcript abundance in colonic samples (36 normal and 48 tumour samples). **(C)** Comparative human and mouse NLRP6/Nlrp6 expression across gastrointestinal segments, integrating a human protein expression overview (accessed December 12, 2025) and publicly available murine expression resources (accessed December 12, 2025), and illustrating broader intestinal expression in mice than is currently suggested by available human datasets (27). **(D)** Schematic summary of increased inducible tumourigenesis in Nlrp6^−/−^ mice compared with wild-type controls in inflammation-driven colorectal cancer models. **(E)** Future biomarker-driven proof-of-mechanism framework in which PPARγ agonism is considered a possible experimental route to explore epithelial NLRP6 induction, while recognising that any association between thiazolidinedione exposure and lower CRC risk is likely to involve additional NLRP6-independent metabolic and immunological effects (9). **(F)** Working diet–microbiota–metabolite model in which microbial short-chain fatty acids may modulate NLRP6-associated epithelial IL-18 signalling and barrier function within a broader metabolic and immunological landscape; this panel is intended as a hypothesis-generating framework rather than a definitive mechanistic pathway. This figure is intended as an integrative, hypothesis-generating summary that combines reported observations with proposed conceptual links; causal relationships should only be inferred where they are explicitly supported by the cited experimental evidence.

Public human tissue resources, including The Human Protein Atlas (14) (**Figure 1A**), provide a high-quality public reference compatible with enriched epithelial NLRP6 signal in the small intestine relative to several other digestive segments. However, because protein-level interpretation of NLRP6 remains technically challenging and requires orthogonal validation using complementary protein-level and spatial approaches, these data should be interpreted as supportive and hypothesis-generating rather than as standalone protein validation. In our local colonic cohort, bulk mRNA analysis of 36 normal and 48 tumour colonic samples showed very low *NLRP6* transcript abundance (**Figure 1B**). This finding supports low bulk transcript detectability in the analysed colonic samples, but does not exclude rare epithelial expression, protein-level activity, or context-dependent functional signalling.

In addition, our exploratory reanalysis of an independent public human intestinal epithelial single-cell RNA-seq dataset (15) showed a descriptive pattern compatible with higher NLRP6 expression in the small intestine than in the colorectum (**Supplementary Data Sheets 1**, **2**). *NLRP6*-positive epithelial cells were more frequent in the small intestine (4.08%) than in the colon and rectum (0.08%), with a marked decline after the ileocecal valve. Because this analysis was intended as exploratory and hypothesis-generating, and because formal donor-level or mixed-model analyses would be required for definitive statistical inference, these single-cell observations are presented as supportive descriptive evidence rather than as formal validation of segmental enrichment.

Additionally, Bracey et al. (16) reported marked ileal enrichment of *NLRP6* expression, whereas colonic expression was not detected in their analysed screening biopsies, further supporting tissue-specific regulation of NLRP6 in the human intestine. Consistent with these observations, a recent mechanistic study (13) did not detect NLRP6 expression in the analysed human colonic cell lines, although this should be interpreted cautiously because cell lines do not necessarily recapitulate the expression landscape of primary human tissue. Collectively, these findings suggest that, in humans, NLRP6 is unlikely to be broadly expressed across the colonic epithelium and may be preferentially associated with the small intestinal epithelium, consistent with region-specific functions in mucosal homeostasis and host–luminal interactions. However, these observations should not be interpreted as definitive evidence of complete absence of NLRP6 from the human colon. Rather, they provide preliminary support for segmental enrichment that now requires orthogonal validation at protein, spatial, and single-cell resolution. In CRC, limited human observational data have suggested a possible association between reduced epithelial NLRP6 protein expression, low IL-18, and more advanced disease; however, these findings remain preliminary and require independent validation and further substantiation (17). Consistent with this context-dependent interpretation, reduced jejunal NLRP6 expression in humans has also been associated with increased intestinal permeability and inflammatory alterations in obesity with type 2 diabetes, further supporting a link between NLRP6, barrier dysfunction, and mucosal inflammatory status (18).

### NLRP6-mediated epithelial defence, mucosal immune surveillance, and CRC-relevant tumour restraint

The putative tumour-protective role of NLRP6 should be understood within the broader framework of epithelial innate immunity and mucosal immune surveillance. Rather than acting as a classical cell-autonomous tumour suppressor, NLRP6 may influence tumour occurrence indirectly by preserving epithelial barrier integrity, supporting IL-18-associated immune signalling, limiting chronic mucosal inflammation, and shaping host–microbiota interactions that condition the local immune microenvironment.

Murine studies support the biological relevance of Nlrp6, but they should not be assumed to directly mirror human segment-specific intestinal biology. Whereas mice show broader intestinal Nlrp6 expression (5, 7), current human datasets discussed above suggest a more restricted pattern. Thus, mouse models are particularly informative for mechanistic insights related to epithelial repair, barrier maintenance, and IL-18-linked tumour restraint (5, 7, 10, 19), while direct validation of segmental expression and function requires human tissues (**Figure 1C**).

*Nlrp6*-deficient mice (Nlrp6^−/−^) exhibit exaggerated colitis and increased susceptibility to inflammation-driven colon tumourigenesis in standard dextran sodium sulphate and azoxymethane/dextran sodium sulphate models, indicating that the loss of NLRP6 facilitates colonic carcinogenesis *in vivo (*7, 10, 20) (**Figure 1D**).

Rosiglitazone, a PPARγ agonist, is discussed here only as a possible tool for future biomarker-driven proof-of-mechanism testing to explore whether epithelial NLRP6 can be induced in the colorectum. An epidemiological study has shown that patients treated with rosiglitazone had a significantly reduced risk of CRC (9), consistent with pharmacological engagement of a putative protective epithelial pathway in the human colon. However, any protective association between thiazolidinedione exposure and reduced CRC risk is also likely to reflect additional metabolic, immunological, anti-inflammatory, and differentiation-related effects beyond NLRP6 alone (**Figure 1E**).

Finally, microbiota-derived short-chain fatty acids (SCFAs) provide a broader metabolic and barrier-related context for this hypothesis, but should not be interpreted as evidence of a specific SCFA–NLRP6 axis in humans. In murine models, altered SCFA availability has been linked to impaired epithelial barrier integrity, reduced Nlrp6-associated inflammasome signalling, and decreased IL-18 production (21). In humans, a systematic review and meta-analysis showed lower faecal concentrations of acetate, propionate, and butyrate in individuals at high risk of CRC and in patients with established disease than in healthy controls (22). These data support an inverse association between SCFA availability and colorectal carcinogenesis, but they remain associative and may reflect multiple NLRP6-independent metabolic, inflammatory, microbial, and epithelial mechanisms. Thus, SCFAs are best viewed here as modulators of the broader mucosal barrier and immune landscape rather than as direct evidence for an NLRP6-specific tumour-protective pathway (**Figure 1F**).

## Discussion

Taken together, the available literature is consistent with the hypothesis that NLRP6 may function as one component of a broader epithelial defence network in the gut, operating through a hierarchical programme rather than through multiple equivalent mechanisms. Within the NLRP6-centred component of this model, the putative primary axis is epithelial NLRP6–caspase-1–IL-18 signalling, which may preserve barrier integrity and limit injury-associated regeneration, thereby reducing conditions that favour tumour initiation or promotion (7, 8, 10, 17). In support of this hierarchical view, recent data in human intestinal epithelial cells indicate that NLRP6 activation may be triggered by endolysosomal damage and loss of vacuolar integrity, rather than primarily by previously proposed pathogen-associated ligands such as dsRNA or lipoteichoic acid (13). Processes such as mucus secretion, epithelial renewal, and microbiota shaping are best interpreted as downstream or closely linked effects within this broader epithelial defence framework (4, 7, 11, 12). By contrast, SCFAs, inflammatory tone, and PPARγ agonism are more appropriately viewed as modulators of NLRP6 activity rather than as independent core mechanisms (9, 18, 22, 23). Importantly, these diet–microbiota–metabolism interactions are also known to influence colorectal carcinogenesis through multiple NLRP6-independent pathways, and the present model should therefore be interpreted as one plausible epithelial defence component within a broader and more complex network. In humans, the currently available data are more consistent with a context-dependent model than with a simple binary presence-versus-absence pattern (14, 16, 17). Within this framework, NLRP6-dependent signalling may contribute to barrier maintenance, mucus secretion, microbiota regulation, and injury control, thereby creating conditions less permissive for chronic mucosal damage and tumour promotion, alongside other established epithelial, microbial, metabolic, and environmental determinants of intestinal carcinogenesis (3, 4, 6, 11, 12). These epithelial effects are immunologically relevant because persistent barrier disruption, altered microbial sensing, impaired IL-18 signalling, and unresolved mucosal inflammation can collectively favour a tumour-promoting immune microenvironment.

Overall, we propose NLRP6 as a testable candidate component of a segment-restricted epithelial defence network in the human gut, rather than as an established tumour-suppressive mechanism. The purpose of this Hypothesis & Theory article is therefore not to assign a solitary causal role to NLRP6, but to integrate the currently available evidence into a testable model that can be refined, validated, or rejected by future human studies. Within this broader multifactorial context, the current evidence is biologically coherent but remains incomplete: human atlas data, single-cell analyses, and our colonic cohort are suggestive, independent human studies indicate limited protein-level and clinicopathological relevance in specific colonic contexts, and murine models support mechanistic plausibility without resolving human segmental biology. The most important next steps are independent human validation, orthogonal protein confirmation, and spatially resolved analyses linked to inflammatory status, dysplasia, and clinical outcome. Clarifying these points will determine whether NLRP6 is primarily a marker of epithelial state, a context-dependent mediator of barrier defence, or a tract-segment-restricted contributor to cancer susceptibility.

To avoid overinterpretation, the present model should be read as an evidence hierarchy rather than as a consolidated human pathway. Murine studies provide established mechanistic plausibility in inflammation-associated colonic tumourigenesis; public human resources, our local colonic cohort, and exploratory single-cell analyses provide supportive but non-definitive observations; and the proposed link between regional NLRP6 enrichment, inflammasome competence, IL-18-associated signalling, epithelial repair, and tumour restraint remains a mechanistic inference requiring direct human validation.

### Future translational testing: a staged validation framework

If future studies support the hypothesis that NLRP6 contributes, alongside other epithelial defence mechanisms, to CRC-relevant mucosal immune surveillance and tumour restraint in the human gut, the most appropriate next step would not be an immediate intervention, but a staged validation programme linking human tissue studies to biomarker-guided proof-of-mechanism testing. In a first phase, the hypothesis should be tested in observational human cohorts by assessing NLRP6 status in normal mucosa, precursor lesions, and established tumours across relevant gastrointestinal segments. Archived and prospective biopsies from selected high-risk gastrointestinal settings could be analysed using standardised transcript-based assays together with orthogonal protein-level approaches and, where feasible, spatial readouts. These studies should determine whether reduced epithelial NLRP6 expression is associated with dysplasia grade, inflammatory activity, segmental location, or risk of progression within a broader multifactorial epithelial defence context.

In a second phase, a short-window, biomarker-driven proof-of-mechanism study should evaluate whether pharmacological activation of PPARγ (24) could be used to explore whether epithelial NLRP6 can be induced in the colorectum. Rosiglitazone is a rational candidate because PPARγ agonism represents a plausible route to explore whether epithelial NLRP6 can be induced in the colorectum, although any potential protective association is also likely to reflect additional metabolic, immunological, and differentiation-related effects beyond NLRP6 alone (9, 23). High-risk patients undergoing scheduled endoscopic surveillance could undergo paired mucosal sampling before and after short-term exposure, with NLRP6 expression and downstream epithelial IL-18/caspase-1-associated signatures used as markers of on-target engagement. Barrier-associated proteins, mucosal inflammatory state, and microbiota/short-chain fatty acid profiles could be incorporated as secondary mechanistic readouts. Demonstration of reproducible NLRP6 induction in human mucosa would provide an important mechanistic bridge between observational pathology and any future preventive intervention strategy.

Only if these earlier stages support reproducible human target validation and on-target pharmacodynamic activity could a longer interventional prevention study be considered as a future conceptual possibility. Such a study would not be justified at the current evidentiary stage, but could eventually be designed in selected high-risk settings, such as hereditary or sporadic polyposis, with standardised colonoscopic surveillance and paired biopsy collection from normal-appearing mucosa and resected polyps. Potential endpoints could include adenoma burden, incidence, and histological progression, with epithelial NLRP6 induction incorporated only as an exploratory biomarker or secondary mechanistic endpoint. Secondary analyses should account for baseline polyp load, aspirin or NSAID exposure, and germline aetiology, including FAP, MAP, and serrated polyposis syndrome. Because thiazolidinediones carry recognised cardiovascular and metabolic risks, safety monitoring and feasibility assessment would be integral to trial design. Within this staged framework, any prevention study should be viewed not as an immediate translational proposal, but as a distant and conditional extension of a human validation pathway.

### Falsifiable predictions and validation criteria

The proposed model is falsifiable. It would be weakened or refuted if donor-adjusted analyses fail to confirm regional enrichment of epithelial NLRP6; if orthogonally validated protein-level or spatial assays do not detect epithelial NLRP6 in the predicted intestinal compartments; if NLRP6 status shows no relationship with inflammatory activity, dysplasia, lesion progression, or IL-18/caspase-1-associated epithelial signatures; or if NLRP6-specific disruption in relevant human epithelial models does not alter barrier, inflammatory, or tumour-promoting phenotypes. These criteria are intended to distinguish a testable hypothesis from a consolidated tumour-suppressive mechanism.

### Limitations

This work has several limitations that should be acknowledged explicitly. First, this is a hypothesis-driven Hypothesis & Theory article and therefore does not provide definitive mechanistic proof that NLRP6 may act as a segment-restricted tumour suppressor in the human gut. Accordingly, the present model should not be interpreted as implying that NLRP6 alone explains the marked epidemiological differences between small-intestinal and colorectal carcinogenesis, which are known to involve multiple epithelial, microbial, metabolic, environmental, and immunological determinants. The human evidence discussed here remains suggestive rather than conclusive, as it relies on public tissue-expression resources, exploratory single-cell signals that still require replication in independent cohorts, and our bulk colonic transcript data, without comprehensive orthogonal human validation.

Second, an important limitation is the lack of spatial and cell-type resolution in the currently discussed human evidence. Bulk tissue measurements cannot determine whether NLRP6 is restricted to particular epithelial lineages, varies along the proximal-distal gut axis, or changes according to inflammatory or neoplastic state. This is particularly relevant because recent single-cell analyses of healthy adult human intestine have shown marked regional and lineage-specific differences across the epithelium, indicating that any segmental NLRP6 model should ultimately be tested at single-cell and spatial resolution rather than inferred from bulk tissue averages alone (25).

Third, protein-level interpretation requires caution. Endogenous NLRP6 expression appears to be low in several epithelial settings, and some commercial antibodies may show non-specific reactivity, making immunohistochemical and immunoblot-based conclusions potentially vulnerable to technical artefact. For this reason, future assessment of NLRP6 in human tissues should ideally combine transcript-based analyses with orthogonal protein-validation strategies and careful antibody validation, particularly in settings where endogenous expression is low and immunoreactivity may be difficult to interpret (26).

Finally, although murine studies provide important mechanistic support, their translational interpretation is limited by species differences in NLRP6 regulation and intestinal expression. Mouse models remain informative for biological plausibility, particularly regarding barrier maintenance, IL-18-linked signalling, and inflammation-associated tumourigenesis, but they do not establish that the same segmental pattern or magnitude of effect applies in humans (5, 7, 10, 16). Likewise, the proposed rosiglitazone-based prevention strategy should still be considered conceptual at this stage and would require prior human biomarker validation and proof-of-mechanism studies before a long-term interventional trial could be justified. Moreover, any protective association between thiazolidinedione exposure and reduced colorectal cancer risk is likely to reflect multiple NLRP6-independent metabolic, immunological, and differentiation-related effects, and should not be interpreted as evidence of a primarily NLRP6-driven mechanism.

## Data Availability

The original contributions presented in the study are included in the article/**Supplementary Material**. Further inquiries can be directed to the corresponding authors.
