# Supplementary material for: NLRP6 as a candidate segmental barrier to carcinogenesis in the human gut: a hypothesis
Source: Front Immunol. 2026 Aug 5;17:1823134. doi: 10.3389/fimmu.2026.1823134 (PMC13485738; doi:10.3389/fimmu.2026.1823134)
Supplement: Supplementary file 1 [file DataSheet1.pdf]

Supplementary Methods

Data source and dataset selection

Single-cell RNA sequencing (scRNA-seq) data from the human intestinal tract were obtained from the gut cell atlas reported by Elmentaite et al. (2021) (1). Processed gene expression matrices were downloaded from ArrayExpress (accession: E-MTAB-9543).

The dataset comprises epithelial and non-epithelial cells isolated from multiple anatomical regions of the adult human intestine. For this analysis, only epithelial cells were considered, as annotated by the authors.

Data processing and integration

Normalized gene expression counts in MatrixMarket format were imported into R (v4.x) using the *Matrix* and *data.table* packages. Gene identifiers were provided as Ensembl IDs, and expression values were used as supplied without additional normalization, given that processed normalized matrices were downloaded directly from the public repository.

Cell-level metadata were obtained from the accompanying experimental design file (`ExpDesign-E-MTAB-9543.tsv`) and merged with the expression matrix using unique cell barcodes.

Cells annotated as epithelial (based on the column *"Factor Value[inferred cell type - authors labels]"*) were selected for downstream analyses.

Definition of anatomical regions

Cells were assigned to anatomical regions based on the metadata field 'Sample Characteristic[organism part]'. For comparative analyses, regions were grouped as follows: small intestine (duodenum, jejunum, ileum) and colorectum (caecum, ascending colon, transverse colon, descending colon, sigmoid colon, rectum).

Gene expression analysis

Expression of NLRP6 (Ensembl ID: ENSG00000174885) was extracted from the normalized expression matrix. Due to the sparse distribution of expression values typical of scRNA-seq data, NLRP6-positive cells were defined as those with detectable expression (expression > 0).

To visualize expression distributions, values were log-transformed using a log1p transformation (log(x + 1)).

Statistical analysis

Differences in continuous gene expression between groups were assessed using the Wilcoxon rank-sum test (two-sided), as data did not follow a normal distribution.

Differences in the proportion of NLRP6-positive cells between groups were evaluated using Fisher's exact test.

All statistical analyses were performed in R, and p-values < 0.05 were considered statistically significant. All figures were generated using the ggplot2 package.

Results

Regional distribution of NLRP6 expression in intestinal epithelial cells

Analysis of single-cell RNA sequencing data revealed that NLRP6 expression was highly sparse across epithelial cells, with the majority of cells lacking detectable expression and a small subset exhibiting high transcript levels.

When stratified by anatomical location, NLRP6 expression was significantly higher in epithelial cells from the small intestine than in the colorectum (Wilcoxon rank-sum test, $p = 2.05 \times 10^{-42}$). This difference was also reflected in the proportion of NLRP6-positive cells, with 4.08% of epithelial cells in the small intestine expressing NLRP6 compared with 0.08% in the colorectum (Fisher's exact test, $p = 3.92 \times 10^{-41}$). These findings indicate that NLRP6 expression is not broadly distributed across colonic epithelium, but instead shows marked segmental restriction within the human intestinal epithelial compartment.

To further refine this regional pattern, NLRP6-positive epithelial cells were also examined across consecutive anatomical segments of the intestinal tract.

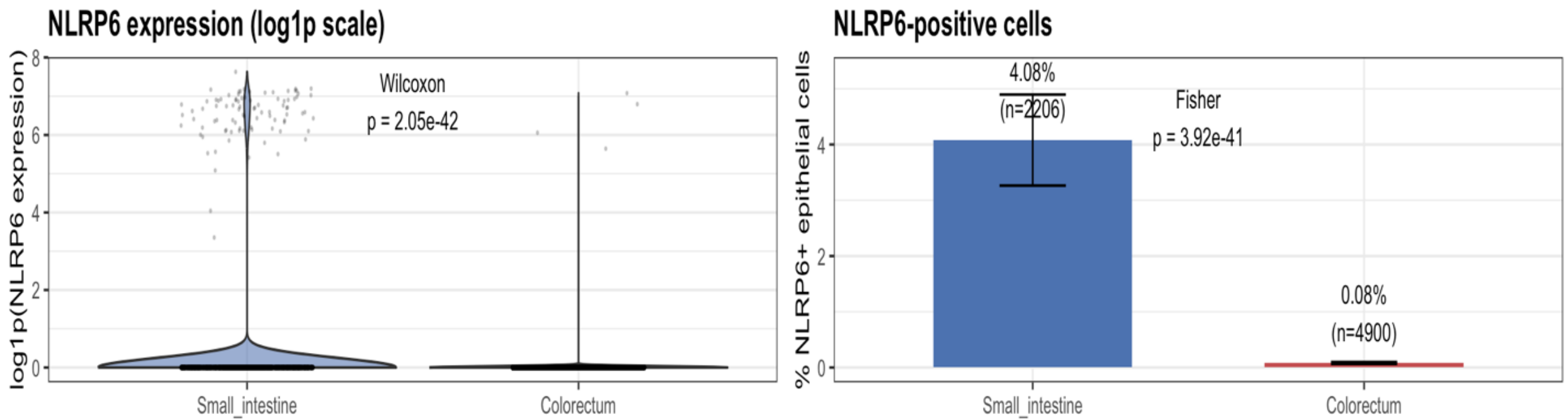

Supplementary Figure 1. NLRP6 expression in epithelial cells across the intestinal axis. (A) Distribution of log1p-transformed NLRP6 expression in epithelial cells from the small intestine and colorectum. Statistical significance was assessed using the Wilcoxon rank-sum test. (B) Percentage of NLRP6-positive epithelial cells in each anatomical group. Differences in the proportion of positive cells were assessed using Fisher's exact test. Sample sizes are indicated for each group.

To explore the transcriptional landscape of epithelial cells expressing NLRP6, dimensionality reduction was performed using Uniform Manifold Approximation and Projection (UMAP). Normalized gene expression counts were log-transformed using log1p, highly variable genes were selected, and principal component analysis was used to generate the low-dimensional embedding. UMAP plots were then used to visualise the spatial distribution of NLRP6-expressing epithelial cells across intestinal regions.

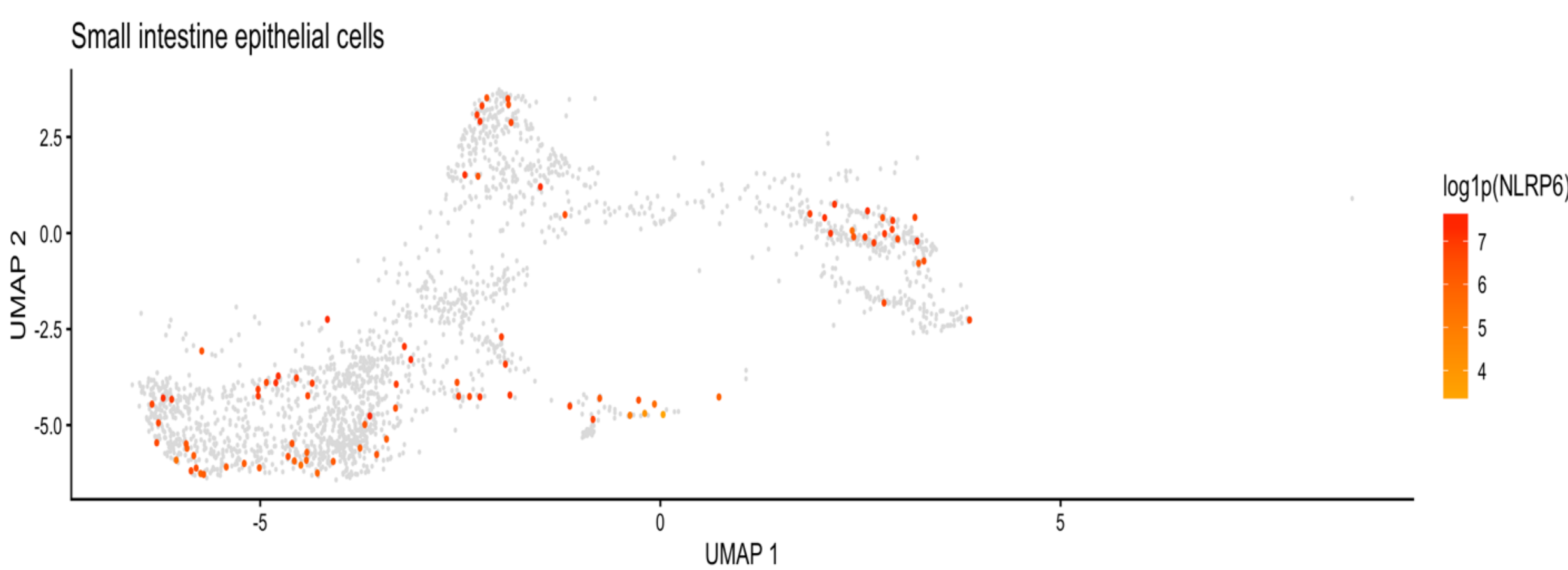

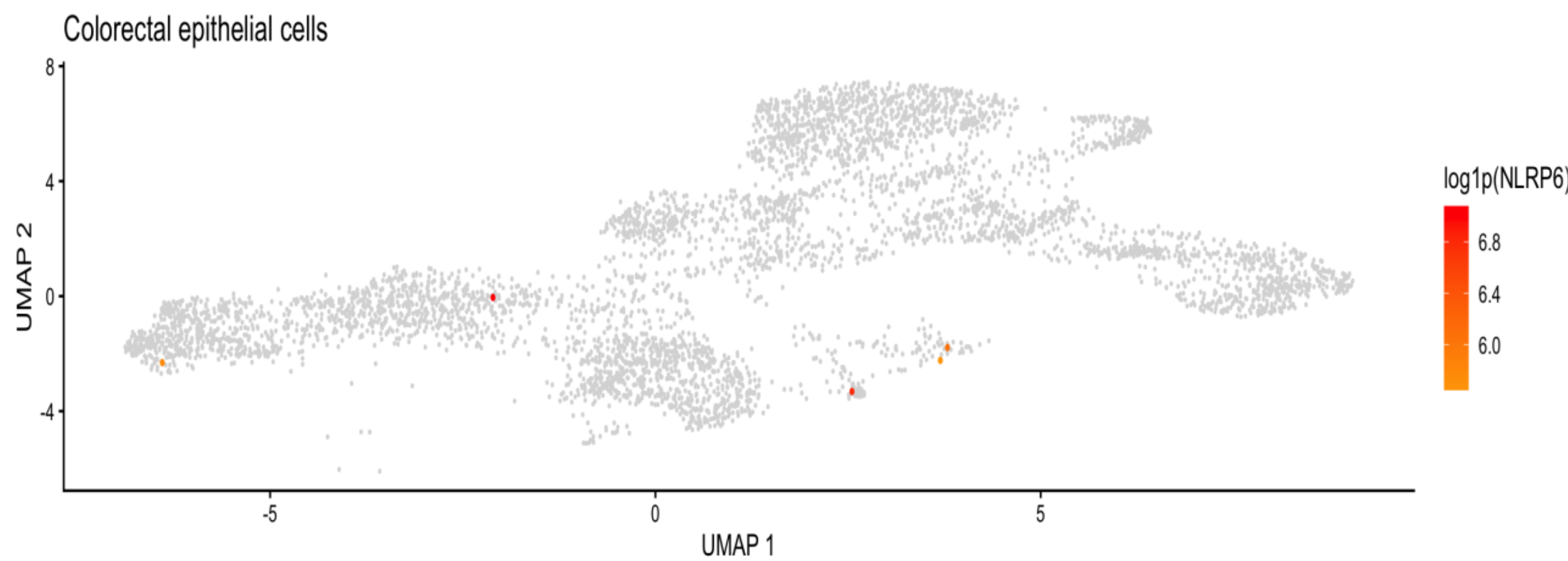

Supplementary Figure 2. UMAP embedding of epithelial cells from the small intestine (upper panel) and colorectum (lower panel), highlighting NLRP6-expressing cells according to log1p-transformed expression values.

Further analysis along the intestinal axis demonstrated a non-uniform regional distribution, with enrichment of NLRP6-positive cells in small intestinal segments, particularly in distal regions, whereas expression remained minimal across all colonic compartments. An abrupt decrease in NLRP6 expression was observed distal to the ileocecal junction, supporting a marked transition in epithelial NLRP6 distribution between the small intestine and colorectum.

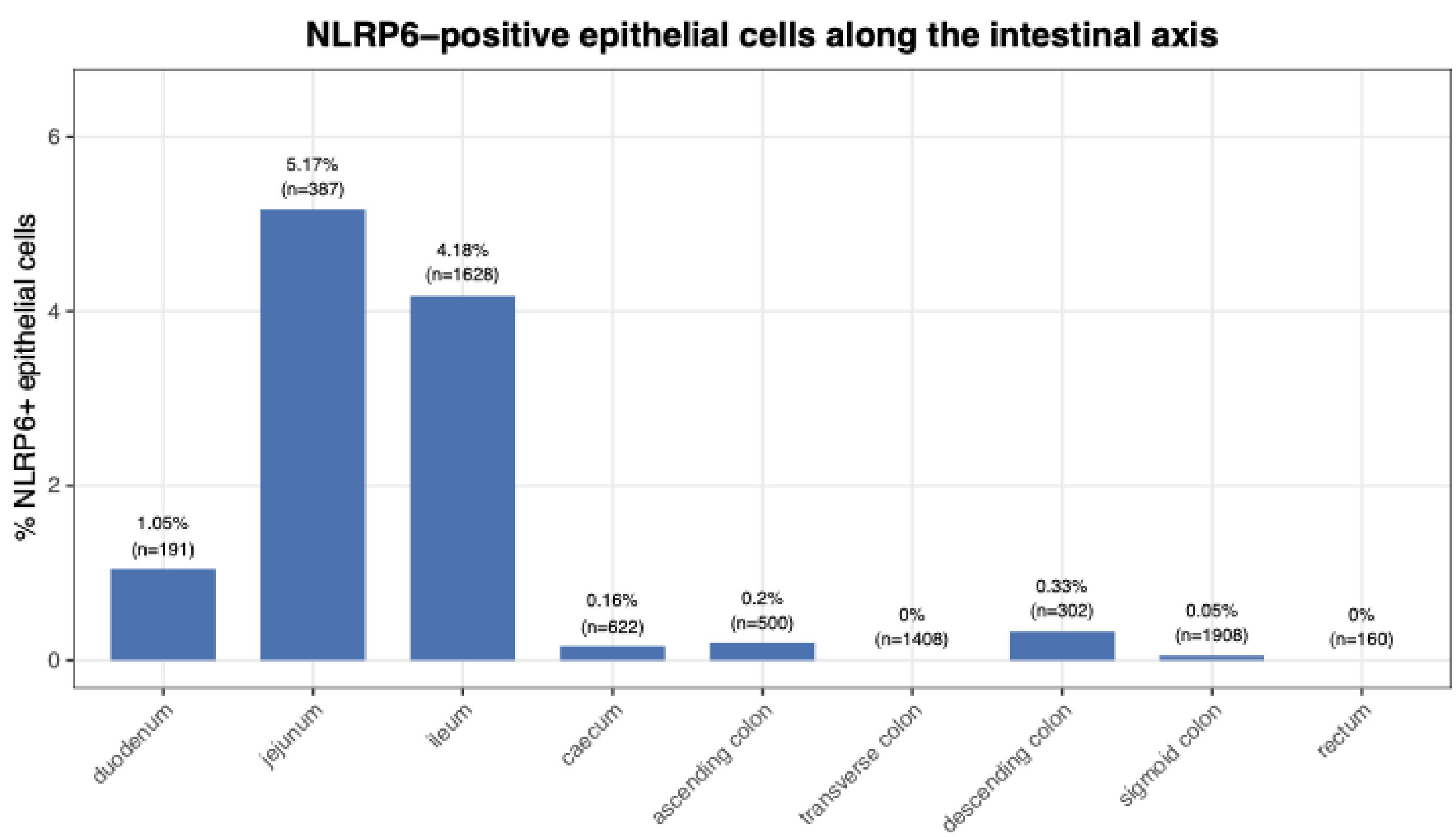

Supplementary Figure 3. Percentage of NLRP6-positive epithelial cells across consecutive anatomical segments of the intestinal tract. The number of analysed cells for each segment is indicated in parentheses.

References

1. Elmentaite R, Kumasaka N, Roberts K, Fleming A, Dann E, King HW, Kleshchevnikov V, Dabrowska M, Pritchard S, Bolt L, et al. Cells of the human intestinal tract mapped across space and time. *Nature* (2021) 597:250–255. doi: 10.1038/s41586-021-03852-1
